# Sensitivity of the dipstick in detecting bacteremic urinary tract infections in elderly hospitalized patients

**DOI:** 10.1371/journal.pone.0187381

**Published:** 2017-10-31

**Authors:** Zvi Shimoni, Joseph Glick, Vered Hermush, Paul Froom

**Affiliations:** 1 Department of Internal Medicine B, Sanz Medical Center, Laniado Hospital, Netanya, Israel; 2 Ruth and Bruce Rappaport School of Medicine, Haifa, Israel; 3 Central Laboratory, Sanz Medical Center, Laniado Hospital, Netanya, Israel; 4 Department of Geriatrics, Sanz Medical Center, Laniado Hospital, Netanya, Israel; 5 Department of Clinical Utility, Sanz Medical Center, Laniado Hospital, Netanya, Israel; 6 School of Public Health, University of Tel Aviv, Ramat Aviv, Israel; University of North Texas, UNITED STATES

## Abstract

**Background:**

The sensitivity of the dipstick in elderly patients with a suspected urinary tract infection (UTI) is unclear because of the inclusion of patients with urine contamination or asymptomatic bacteriuria in previous studies.

**Methods:**

We selected consecutive patients aged 65 years or older hospitalized in internal medicine departments with bacteremic UTI (same organism in blood and urine cultures) minimizing misclassifications. The false positive rate was determined in consecutive patients with negative culture results. A positive dipstick was a test result with a trace leukocyte esterase and/or nitrite positivity. Bacteriuria was the growth of at least 10^5^ colony-forming units per milliliter of urine.

**Results:**

Of 20,555 consecutive patients, 228 had a bacteremic UTI, and 4069 a negative culture result. The sensitivity of the dipstick was 96.9% (95% CI—93.7–98.6) with a false positive rate of 42.4% (95% CI, 41.0–43.8) in those with a negative culture result.

**Conclusions:**

In elderly hospitalized patients with a bacteremic UTI, the dipstick urinalysis is highly sensitive, much higher than reported previously in studies of UTIs in the elderly. It is unclear whether the observed high sensitivity of the dipstick was due to the exclusion of patients with asymptomatic bacteriuria or to spectrum bias. Studies of the clinical utility/disutility of using a negative dipstick to rule out a urinary tract infection are warranted.

## Introduction

A positive urine culture may be due to contamination, asymptomatic bacteriuria or a symptomatic urinary tract infection (UTI) [1]. In hospitalized elderly patients, it is unclear when to suspect a symptomatic UTI, and when to initiate treatment with antibiotics. This is because geriatric patients with a symptomatic UTI usually have nonspecific systemic symptoms [1, 2, 3, 4] and there is a high prevalence of asymptomatic bacteriuria [4, 5, 6, 7] that doesn't require antibiotic therapy. Furthermore, the high prevalence of pyuria in the absence of a UTI [1, 7, 8] limits a positive test's value in establishing the diagnosis.

On the other hand, there might be a role for the dipstick, microscopic urinalysis, and/or automated cell counters to rule out a UTI, limiting the need for hospitalization, early antibiotic therapy, urine cultures [9,10] and lowering the risk of inappropriate antibiotic therapy in those with asymptomatic bacteriuria. This might also identify septic patients with an extra-urinary tract infection who require further diagnostic studies.

In order to decrease unnecessary treatment of those with asymptomatic bacteriuria, some experts consider pyuria (>10 white blood cells per high power field (WBC/HPF) on microscopic urinalysis as a necessary component of any UTI definition [1, 11]. The ability of the dipstick and microscopic urinalysis to rule out a urinary tract infection however, is unclear due in part to the nonselective inclusion criteria of previous studies and the lack of a gold standard for the diagnosis of a UTI. Over diagnosis occurs in patients hospitalized for reasons outside the urinary tract with asymptomatic bacteriuria. Under diagnosis could occur if the requirement of >10 WBC/HPF on a microscopic urinalysis is not sensitive enough.

Bacteremic urinary tract infections represent a unique and desirable condition in which to assess the sensitivity of the urinalysis. Infection with the same organism in the blood and urine renders contamination or asymptomatic bacteriuria unlikely, minimizing misclassification [12].

The aim of this study was to determine the sensitivity and specificity of the dipstick and reflex microscopic urinalysis to detect a bacteremic UTI in a large cohort of hospitalized patients 65 years or older. We hypothesized that sensitivity of the dipstick will be high in those with a bacteremic UTI, and that a reflex microscopy requiring >10 WBC/HPF for the diagnosis of a UTI will be insensitive.

## Methods

We retrospectively selected all patients aged 65 years or older, hospitalized in internal medicine departments from January 1, 2015 until December 31, 2016 who had a urinalysis and urine culture done on admission. From this group we selected two groups; one with a negative urine culture and the other with a bacteremic urinary tract infection defined as growth of the same bacteria on both urine and blood cultures. Information extracted from the computerized charts included age, sex, chief complaints [13], urine and blood cultures, urinalysis results, complete blood counts, serum albumin, serum creatinine values, and blood glucose levels.

The Siemens healthcare diagnostics reagent strips for urinalysis (Siemems Healthcare Diagnostics LTDD, Camberley UK), analyzed on the Clinitek Advantus (Siemens Healthcare Diagnostics Tarrytown NY, USA) tested for nitrites (positive or negative) and leukocyte esterase (trace value considered positive) predetermined to maximize sensitivity. A positive dipstick was a test result with a trace leukocyte esterase and/or nitrite positivity. For quality control, the laboratory tested daily positive and negative controls, and twice yearly participated in an external quality control program (College of American Pathologists External Quality Assurance Proficiency Testing). All results were within consensus values. For any positive findings (nitrites positive, ≥ trace positive leukocyte esterase, or trace hemoglobin), a reflex microscopic analysis quantified the number of white blood cells per high-powered field (WBC/HPF) [14]. Over a 2-week period, we did selective test-retests for leukocyte esterase (LE) and nitrite samples sent for urinalysis. All 20 nitrite positive and 20 nitrite negative test results were the same on retesting. Forty negative LE tests were negative on retesting, and 38 of 40 trace positive tests were positive on retesting consistent with results reported previously using a different analyzer [15].

The laboratory technician was aware of the dipstick result when performing the reflex microscopic urinalysis. For the results of the microscopic urinalysis, we considered >3 WBC/HPF as one cut-off value used by others [12] and consistent with a trace positive leukocyte esterase value (median = 3 WBC/HPF (1^st^-3^rd^ quartiles- 2–7, N = 702) and >10 WBC/HPF for the second cut-off value thought to be a necessary component of any UTI definition by some experts [1, 11]. In order to maximize the possible utility of the microscopic urinalysis, we considered all missing values to be negative in those with a negative urine culture and positive in those with a bacteremic UTI (i.e. best-case scenario).

Cultures were processed using standard microbiologic methods, and isolates were identified by the VITEK 2 system (bioMérieux, Marcy l’Etoile, France). The technicians were unaware of the urinalysis test results. Bacteriuria was defined as the presence of at least 10^5^ colony-forming units per milliliter of urine. We reviewed the charts of patients with a bacteremic UTI but negative dipstick to determine if there would be disutility in assuming that they did not have a UTI.

Median and quartiles values were calculated for continuous variables, and proportions for discrete variables as well as 95% confidence intervals (CI). For continuous variables, we determined statistically significant differences using the median test. For discrete variables, we used the two-tailed chi-squared test to compare differences between the groups. A p value of < 0.05 was considered significant.

The study received approval from the Laniado Hospital Ethics Committee (0054-17-LND). The ethics committee waived the need for patient consent. The Israel Ministry of Health reviews local ethics committee decisions.

## Results

There were 20,555 patients extracted from the database, and 39.1% (N = 8040) had a dipstick done on admission of whom 88.7% (N = 7129) had a concomitant urine culture result despite a negative dipstick in 42.3% (N = 3401) of the patients. Of those with a urinalysis and culture results, 4609 had a negative urine culture, and 228 patients had positive blood and urine cultures with the same organism; 171 (75.0%) had Escherichia coli, 25 (11.0%) Klebsiella pneumonia, 11 (4.8%) Proteus species, and the other 21 (9.2%) had either citrobacter (N = 6), Staphylococcus aureas (N = 4), Pseudomonas (N = 3), Enterococcus faecalis (N = 3), Streptococcus anginosis (N = 2), Providentia stuarti (N = 2), Actinetobacter baumannii (N = 1), Enterobacter cloace (N = 1).

Compared to those with negative cultures, the patients with a bacteremic UTI had a significantly higher proportion of females, and lower hemoglobin, albumin, and higher serum creatinine values Table 1. Their white blood cell counts and neutrophil counts were higher and they had a higher proportion of patients presenting with fever. The death rates were nearly identical in the two groups.

**Table 1:** Comparison of patients with negative urine cultures to those with a bacteremic urinary tract infection.

Test	Negative N = 4609 Median (1^st^ -3^rd^ quartile)	Bacteremic UTI N = 228 Median (1^st^ -3^rd^ quartile)	P values
Age	85(78–90)	85 (79–91)	0.452
Albumin(mg/dL)*	3.6 (3.1–4.0)	3.4 (3.0–3.8)	< 0.001
Creatinine(mg/dL)	1.1 (0.8–1.5)	1.2 (0.9–1.7)	< 0.001
Hemoglobin (g/L)*	120(107–133)	117(105–130)	0.054
WBC x 10^9^/L*	10.2(7.6–13.9)	11.8(8.8–15.8)	< 0.001
Neutrophils x10^9^/L*	7.9 (5.5–11.7)	10.6(7.5–14.4)	< 0.001
Hospitalization (days)	5 (3–8)	6 (5–10)	< 0.001
Non parametric	N (%)	N (%)	
Female	2299 (49.9)	133 (58.3)	< 0.001
Fever	1508 (32.7)	134 (58.8)	< 0.001
Death	513 (11.1)	25 (11.0)	0.938

*few missing values

The sensitivity of a trace LE for a bacteremic UTI was 96.1% (95% CI, 92.3–98.2) and increased slightly with the addition of nitrite positivity (96.9%, 95% CI—93.7–98.6) Table 2. False positives were 42.4% (95% CI, 41.0–43.8) in those with a negative urine culture.

**Table 2:** Trace leukocyte esterase and nitrite positivity in those with negative urine cultures and those with a bacteremic urinary tract infection.

Findings	Negative Total = 4609* N (%, 95% CI)	Bacteremic UTI Total = 228** N (%, 95% CI)
1 Leukocyte esterase-trace	1860 (40.4, 39.0–41.8)	219 (96.1, 92.3–98.2)
2 Nitrite positive	632 (13.7, 12.8–14.7)	144 (63.2, 56.7–69.2)
1 or 2	1954 (42.4, 41.0–43.8)	221 (96.9, 93.7–98.6)
Reflex >3 WBC/HPF	1613 (35.0, 33.6–36.4)	212 (93.0, 88.8–95.7)
Reflex >10 WBC/HPF	928 (20.1, 19.0–21.3)	178 (78.1, 72.2–83.0)***

* there were 1217 tests with missing microscopy, assumed to be negative

**there were 6 missing tests assumed to be positive for best case scenario.

*** p < 0.001 compared to either nitrite or leukocyte esterase positive (1 or 2)

There was a reflex microscopic urinalysis result in 78.3% (5688/7264) of the dipstick tests done. For the best-case scenario all missing tests were considered to identify correctly those with negative cultures (N = 1217) and those with a bacteremic UTI (N = 6). Nevertheless, requiring > 10 WBC/HPF for confirmation of a UTI diagnosis decreased the sensitivity to 78.1% (95% CI 72.2–83.0) and false positive tests decreased maximally to 20.1% (95% CI 19.0–21.3)Table 2. At a cut off value of >3 WBC/HPF, there was a trend for a decreased sensitivity even after assuming that the 6 missing values were positive (p = 0.09), with a similar proportion of false positive results.

There was no clinical disutility in assuming the seven patients with a negative urinalysis did not have a urinary tract infection. All presented with fever and would have been given antibiotics anyways, two patients presented with other concomitant sources of their fever (lung infiltrates); one patient presented with seizures, and 3 patients with leukocytosis and >85% neutrophils presented with diarrhea, dysuria and hyperglycemia, and with fever only. Finally, there was one patient who was admitted with a partially treated urinary tract infection whose fever continued despite oral antibiotics. All the patients had urine and blood cultures with E coli sensitive to ceftriaxone the preferred antibiotic given to elderly patients presenting with fever and a suspected bacterial infection.

## Discussion

The major finding in our study is that a positive nitrite and/or trace leukocyte esterase identified 96.9% of hospitalized patients aged 65 years or older with a bacteremic UTI Table 2. This result is much higher than has been reported previously for elder adults with a presumed urinary tract infection [1, 16], where the sensitivity was estimated to be 82% (95% CI, 74%–92%) ([1,16]. We are unaware of previous studies done in adults, but our findings are consistent with those reported in 245 children less than 3 months of age in a multicenter study [12]; the sensitivity was 97.6% for leukocyte esterase alone in detecting a bacteremic UTI.

The strength of this study is that we chose patients with a bacteremic UTI, patients with a high probability of an actual UTI in a well-defined cohort. Previous studies in adults suffer from misclassification bias because of the difficulty in differentiating between asymptomatic and symptomatic bacteriuria. Furthermore, our cohort is typical of patients hospitalized in other internal medicine departments with a predominance of elderly patients, and our methodology is that most commonly used in other medical centers in the United States [12] and elsewhere.

Our study has limitations. First of all this is a single center study with the use of only one analyzer. Our findings require confirmation, and caution in extrapolation to other centers. The performance of various analyzers is variable, and laboratories need to test for precision and correlation of the dipstick with microscopy if they want to determine cut-off values that correspond to our study where the trace leukocyte esterase results had a median value of 3 WBC/HPF on microscopic examination. However, the only similar multicenter trial achieved similar results without harmonizing the dipstick methodology [12] suggesting that our results can be extrapolated to other centers, even without correlating the results with microscopic analysis. Secondly, extrapolation from a bacteremic urinary tract infection to those with isolated bacteriuria might be unwarranted due to spectrum bias; screening tests are more sensitive when the disease is severe. This bias might explain the higher sensitivity of the dipstick found in our study compared to previous studies, but is only likely to decrease the observed sensitivity of reflex testing. Thirdly, we defined bacteriuria as the presence of at least 10^5^ colony-forming units per milliliter of urine, and experts commonly lower the cutoff in patients with urinary tract symptoms that could have lowered the observed sensitivity. However, none of the patients with bacteremia had a urine culture with the same organism at lower concentrations. Fourthly, it is possible that in some cases, the urine cultures might have been positive because of seeding from bacteremia caused by an infection outside the urinary tract. In fact, two of the seven negative dipsticks in patients with a bacteremic UTI had concomitant lung infiltrates. This misdiagnosis bias however, only decreases the observed sensitivity. Fifthly, it is unclear if our results can be extrapolated to younger age groups including pregnant females, patients hospitalized in surgical departments, and intensive care units as well as to non-hospitalized patients. Finally, we did not use automated leukocyte and bacterial cell counters that might improve both sensitivity and specificity. Studies are warranted that compare the ability of various quantitative cell counters to that of nonparametric dipstick test results in identifying patients with a bacteremic UTI.

Our findings suggest that the requirement of either a trace positive leukocyte esterase or a positive nitrite on the dipstick to make the diagnosis of a urinary tract infection might be justified. In the stable elderly patient without directional symptoms, a negative dipstick might influence the decision to observe them as outpatients and withhold antibiotic therapy. Requiring a positive dipstick test result for urine culture implementation as suggested by others [9, 10] would have reduced urine cultures significantly, at the expense of missing 3.1% of patients with a bacteremic UTI. It is reassuring that in patients with a bacteremic UTI we did not find any clinical disutility in those with a negative dipstick, since they would have received upfront antibiotics even if the physician assumed that the patient did not have a UTI. Lower culture rates will lower the risk of inappropriate treatment of patients with asymptomatic bacteriuria. Furthermore, in septic patients, a negative dipstick test result might identify septic patients who require further diagnostic studies to determine the source of their infection. However, before a negative dipstick test result can be used to provide differential care, the clinical utility/disutility of such a policy requires studies of patients with isolated bacteriuria.

Since microscopic urine examinations were only done for those with a positive dipstick, we were only able to calculate the best-case scenario. Yet even after assuming that all missing tests identified correctly those with and without a bacteremic UTI, our findings do not support the recommendations for reflex microscopic urinalysis testing in those with a positive dipstick and the requirement of >10 WBC per HPF for the confirmation of a UTI diagnosis [1]. Such a requirement would lower the sensitivity for a bacteremic UTI to 78.1% or less. Specificity was increased but a positive test result is unlikely to influence clinical decisions in the patient with a suspected UTI because the prevalence of > 10 WBC/HPF in patients with negative culture results was still around 20% even after assuming that a large number of missing cases were negative. Decreasing the cutoff to > 3 WBC/HPF increased sensitivity but still resulted in an increased number of bacteremic UTI patients with negative test results. Reflex microscopic testing after a positive dipstick test result is still a common practice [17], but our results support others who have stopped reflex microscopic examination in outpatients [18, 19] and inpatients [19] only done by physician request. On the other hand, there might be a role for microscopic examination in those with a negative dipstick in order to increase sensitivity. In any case, because of poor sensitivity, the microscopic urinalysis should not be used to confirm the need for urine cultures or antibiotic therapy.

We conclude that a dipstick urinalysis can detect nearly all elderly patients with a bacteremic UTI but the reflex microscopic urinalysis to make the diagnosis of a UTI lead to a significant decrease in sensitivity Table 2. The clinical utility and disutility of requiring either a trace leukocyte esterase or positive nitrite test result to make the diagnosis of a UTI is uncertain but deserves consideration. Such a policy would lower urine culture rates, lower the risk of inappropriate antibiotic therapy in patients with asymptomatic bacteriuria, and might identify septic patients who require further diagnostic studies to determine the source of their infection.

## Supporting information

S1 File Minimal data xls file. (XLS)
